# Measuring functional connectivity using MEG: Methodology and comparison with fcMRI

**DOI:** 10.1016/j.neuroimage.2011.02.054

**Published:** 2011-06-01

**Authors:** Matthew J. Brookes, Joanne R. Hale, Johanna M. Zumer, Claire M. Stevenson, Susan T. Francis, Gareth R. Barnes, Julia P. Owen, Peter G. Morris, Srikantan S. Nagarajan

**Affiliations:** a Sir Peter Mansfield Magnetic Resonance Centre, School of Physics and Astronomy, University of Nottingham, University Park, Nottingham, NG7 2RD, UK; b Wellcome Trust Centre for Neuroimaging, University College London, London, UK; c Biomagnetic Imaging Laboratory, Department of Radiology, University of California, San Francisco, USA; d Joint Graduate Group in Bioengineering, University of California, San Francisco and Berkeley, USA

**Keywords:** MEG, fMRI, 7T, Functional connectivity, Neural oscillations, BOLD, Resting state, Coherence, Imaginary coherence, Envelope correlation

## Abstract

Functional connectivity (FC) between brain regions is thought to be central to the way in which the brain processes information. Abnormal connectivity is thought to be implicated in a number of diseases. The ability to study FC is therefore a key goal for neuroimaging. Functional connectivity (fc) MRI has become a popular tool to make connectivity measurements but the technique is limited by its indirect nature. A multimodal approach is therefore an attractive means to investigate the electrodynamic mechanisms underlying hemodynamic connectivity. In this paper, we investigate *resting state* FC using fcMRI and magnetoencephalography (MEG). In fcMRI, we exploit the advantages afforded by ultra high magnetic field. In MEG we apply envelope correlation and coherence techniques to source space projected MEG signals. We show that beamforming provides an excellent means to measure FC in source space using MEG data. However, care must be taken when interpreting these measurements since cross talk between voxels in source space can potentially lead to spurious connectivity and this must be taken into account in all studies of this type. We show good spatial agreement between FC measured independently using MEG and fcMRI; FC between sensorimotor cortices was observed using both modalities, with the best spatial agreement when MEG data are filtered into the β band. This finding helps to reduce the potential confounds associated with each modality alone: while it helps reduce the uncertainties in spatial patterns generated by MEG (brought about by the ill posed inverse problem), addition of electrodynamic metric confirms the neural basis of fcMRI measurements. Finally, we show that multiple MEG based FC metrics allow the potential to move beyond what is possible using fcMRI, and investigate the nature of electrodynamic connectivity. Our results extend those from previous studies and add weight to the argument that neural oscillations are intimately related to functional connectivity and the BOLD response.

## Research highlights

► The utility of beamforming as a source space projection algorithm for use with functional connectivity (FC) measurements is explored. ► Simulations are described and prove to be a robust methodology to eliminate spurious FC from source space MEG measurements. ► The spatial signature of resting state FC between left and right sensorimotor cortices can be measured independently using MEG and fMRI. ► Excellent agreement between motor cortex FC measured using both MEG and fcMRI. ► Multiple MEG FC metrics exploit the direct nature of MEG.

## Introduction

In recent years, the importance of measuring connectivity between spatially separate but functionally related brain areas has become of key interest in the study of brain function. Recruitment of multiple brain regions to form networks is thought to be integral to the way in which the brain processes information (Schnitzler and Gross, 2005; Uhlhaas and Singer, 2010) and abnormal recruitment of brain areas is thought to be implicated in pathologies such as schizophrenia (Friston, 1999; Phillips and Silverstein, 2003; Stephan et al., 2009). The study of functional connectivity (FC) is therefore of great importance to the field of neuroimaging. In most neuroimaging studies, the term functional connectivity has been used to indicate correlation between signals observed in spatially separate brain regions. Much progress in this area has evolved from the study of spontaneous fluctuations in blood oxygenation level dependent (BOLD) functional magnetic resonance imaging (fMRI) signals. Spontaneous BOLD fluctuations are observed during the resting state (Biswal et al., 1995) and superimposed onto task driven responses (Fox et al., 2006). The timecourses of resting state signals from spatially separate areas have been shown to be correlated in time (Biswal et al., 1995), the implication being that activity in these areas is linked, even in the absence of external stimuli. Such measurements have been termed functional connectivity MRI (fcMRI) and a number of data driven analysis techniques (e.g. seed based correlation and independent component analysis) have been applied to fcMRI data revealing the spatial signature of a number of resting state networks (Fox and Raichle, 2007; Laufs, 2008).

Improvements in fcMRI measurement have been shown to result from the use of ultra high (7T) magnetic field. The BOLD response becomes more closely related to microvasculature as field strength is increased (Yacoub et al., 2001). Further, BOLD contrast to noise ratio (CNR) also increases with field and a recent study has shown that at 7T, fcMRI yields network measurements with improved spatial resolution and sensitivity (Hale et al., 2010). Unfortunately, all BOLD measurements (including those at ultra high field) are to some degree confounded since they are indirect assessments of brain activity; they relate to blood flow and not to electrical processes and are therefore limited by poor temporal resolution due to the protracted hemodynamic response. In addition, fcMRI is affected by non-neuronal physiological signals, e.g. the cardiac and respiratory cycles (Wise et al., 2004; Birn et al., 2006, 2008). Such artifacts worsen with field strength and add structured interference to fcMRI, potentially leading to spurious FC. Most importantly, the indirect nature of fcMRI means that the electrical mechanisms that mediate FC cannot be elucidated. As the importance of FC grows, the introduction of non-hemodynamic metrics and our ability to develop a complete understanding of brain network activity and connectivity will become a key goal in neuroscience.

Neural oscillations have an established role in coordinating neural activity both in local networks (Gray et al., 1989; Womelsdorf et al., 2007) and over longer distances (von Stein et al., 2000). Such oscillations are thought to be intimately involved in network activity. Insight into the relationship between oscillatory processes and fcMRI network measurements has been gained from concurrent electroencephalography (EEG) and fMRI (Laufs, 2008). Some studies have shown that spontaneous fluctuations in α (8–13 Hz) band power (measured at an EEG electrode) is negatively correlated with BOLD signal changes in occipital and parietal cortices; other studies have reported positive correlations between α power and BOLD in the thalamus (Goldman et al., 2001; Moosmann et al., 2003; Mantini et al., 2007). Laufs et al. (2003) and Mantini et al. (2007) demonstrated that fronto-parietal network activity is associated with ongoing modulation of α power, implying that a single EEG frequency band can be associated with multiple brain areas. Further, Mantini et al. (2007) have shown that electrical activity in the β band is associated with resting state motor network activity identified using fcMRI. Unfortunately, most EEG/fMRI studies focus on EEG data in sensor space. It is well known that the inhomogeneous conductivity profile in the head means that patterns of electrical potential measured at the scalp are diffuse, and can be distorted. This makes the EEG signal at a single electrode an average of electrical activity across a large volume of tissue. Further, a single sensor most affected by a source doesn't necessarily directly overlay that source. It is therefore difficult to pinpoint the location of electrical oscillators using EEG. Most importantly, it is hard to disentangle two spatially separate sources that may exhibit FC since the same channels can be affected by both sources. Finally, EEG measurements at high magnetic field are limited by poor signal to noise ratio (SNR) and interference caused by the MR scanner. These effects combined limit the utility of concurrent EEG/fMRI.

Magnetoencephalography (MEG) is a non-invasive technology in which the magnetic fields induced by neuronal current flow in the brain are measured above the scalp (Cohen, 1972). MEG has been shown to be an excellent means to measure neural oscillatory processes. Furthermore, unlike electric fields, magnetic fields are not distorted by inhomogeneous conductivity in the head. This, coupled with the high number of sensors (~ 300 in modern systems) and advanced source reconstruction algorithms (Robinson and Vrba, 1998; Zumer et al., 2007; Wipf et al., 2010), makes MEG data more appropriate for projection into source space. MEG studies performed in this way can exhibit vastly improved spatial resolution compared to EEG. For FC measurement, projection is advantageous (Schoffelen and Gross, 2009) since: 1) It limits the effect of field spread (a single source affecting multiple sensors) making results easier to interpret and allowing spatial comparison between FC maps generated independently using fcMRI and MEG; 2) The improved spatial resolution allows separation of signals originating from spatially separate brain locations; and 3) Projection allows for increased signal to noise ratio (SNR) (see for example Brookes et al., 2008a,b, 2009, 2010). These facts make MEG the most attractive non-invasive means to measure electrodynamic FC. However, care must be taken when making these measurements (Schoffelen and Gross, 2009) since the magnetic field induced from a single current source will affect multiple MEG sensors, and the ill posed nature of the inverse problem means that, while spatial resolution is improved compared to EEG, signals originating from spatially separate voxels in source space are not necessarily independent. Cross talk (or signal leakage) between voxel timecourses can therefore generate spurious connectivity measurement. Additionally, MEG is susceptible to interference from environmental noise which may also affect FC metrics.

Despite difficulties a number of studies have employed MEG to measure FC in both sensor and source space and a variety of methodologies have been described. Dynamic imaging of coherent sources (Gross et al., 2001) is an elegant technique in which a frequency domain beamformer is employed to project MEG data; coherence between brain regions is subsequently measured. Other studies (e.g. Guggisberg et al., 2008) have employed time domain beamforming and imaginary coherence (imaginary coherence excludes coherent sources with zero time lag and therefore eliminates the effect of field spread and cross talk). Other metrics include phase lag index (Stam et al., 2007) (which quantifies asymmetry in the phase lag distribution (field spread and cross talk would cause a symmetric distribution and is therefore eliminated)) and synchronization likelihood (Stam and van Dijk, 2002) (which takes two separate electrical signals and looks for isochronous recurrence to a certain part of their (individually different) attractors). Interestingly, in a recent paper (Liu et al., 2010) Liu and colleagues employed a MEG sensor space ‘envelope correlation’ metric to show that inter-hemispheric connectivity (observed by fcMRI) is mirrored by inter-hemispheric neuromagnetic correlation (though no direct comparison was made). In the envelope correlation technique, data are frequency filtered to the band of interest and correlations between power envelopes of oscillatory timecourses from spatially separate brain areas are sought. The findings of Liu et al. are in agreement with papers showing that task driven changes in hemodynamics are related to fluctuations in the envelope of band limited oscillatory power (Singh et al., 2002; Brookes et al., 2005; Stevenson et al., 2011; Zumer et al., 2010). However, since measurements were made in sensor space, the spatial structure of connectivity inferred was unclear. In contrast to sensor space analyses, de Pasquale et al. (2009) used source space reconstructions with minimum-norm techniques and showed that the dorsal attention and the default mode networks, commonly observed using fMRI, are also observable in MEG, particularly using non-lagged correlations in short temporal windows. However, minimum-norm techniques are known to have poor spatial resolution and large reconstruction errors in time-course estimation. Nevertheless, these papers represent some of the first demonstrations of similarity between hemodynamic and electrical resting state FC measurements which we further investigate here.

In this paper, we investigate techniques to measure resting state functional connectivity (*defined as correlation or coherence between signals from spatially separate brain regions*) using MEG. We compare our results to those gained from fcMRI measurements in the same subjects. In fcMRI, we exploit the advantages afforded by ultra high (7T) magnetic field. In MEG, we apply envelope correlation and coherence techniques to brain space reconstructions of neuronal activity generated using adaptive beamformers and examine the relationship between the reconstructed neuronal activity and fcMRI. Our study has three specific aims: 1) To investigate the applicability of beamforming as a source space projection algorithm for FC measurement; 2) To compare multiple MEG metrics including envelope correlation, coherence and imaginary coherence; and 3) To compare the consistency in the spatial signature of motor network FC measured independently using fcMRI and MEG and to test the hypothesis that neural oscillatory processes are intimately related to hemodynamic FC. In what follows, the Methods section describes in detail our processes for measuring FC using MEG data, and testing the validity of those measurements. In the Results section we address individually each of the three aims stated above and we show that MEG represents a useful modality with which to investigate FC, with good agreement between measurements generated using these two disparate modalities. Finally, in the discussion, we examine the nature of the electrical effects that underlie hemodynamic FC.

## Methods

### Data acquisition

Six healthy right handed subjects took part in the MEG experiments. Five of those six subjects took part in the fMRI experiments. The study was approved by the University of Nottingham Medical School ethics committee.

#### MEG

MEG data were recorded using the third order gradiometer configuration of a 275 channel CTF MEG system at a sampling rate of 600 Hz. The scanner is housed inside a magnetically shielded room (MSR) and a 150 Hz low pass anti-aliasing hardware filter was applied. All subjects underwent a single experiment which comprised two contiguous phases, a *resting state* phase and a *localizer* task. During the *resting state* phase, subjects were asked to lie in the scanner with their eyes open and relax while 300 s of resting state data were acquired. The *localizer* task comprised a total of 10 trials. A single trial comprised 30 s of left index finger movement, 30 s of right index finger movement, 30 s during which both left and right index fingers were moved together, and 30 s of rest. The movement itself comprised abductions and extensions of the index finger. The motor task was cued visually via projection through a waveguide in the MSR onto a back projection screen located 40 cm in front of the subject. During data acquisition the location of the subject's head within the scanner was measured by energizing coils placed at 3 fiducial points on the head (nasion, left preauricular and right preauricular). If any subject moved more than 5 mm during the experiment, data from that subject was discarded. Following data acquisition, the positions of the coils were measured relative to the subject's head shape using a 3D digitizer (*Polhemus isotrack*). An MPRAGE structural MR image was acquired using a Philips Achieva 3T MRI system (1 mm^3^ isotropic resolution, 256 × 256 × 160 matrix size, TR = 8.3 ms, TR = 3.9 ms, TI = 960 ms, shot interval = 3 s, FA = 8° and SENSE factor = 3). The locations of the fiducial markers and MEG sensors with respect to the brain anatomy were determined by matching the digitized head surface to the head surface extracted from the 3T anatomical MRI.

#### fMRI

fMRI data were acquired using a Philips Achieva 7T system. All subjects underwent a *resting state* and a *localizer* experiment (note, these data have previously been published in a study by Hale et al. (2010) comparing 3T and 7T fcMRI). In the *resting state* experiment, subjects were asked to lie in the scanner with their eyes open and relax while 300 s of BOLD data were acquired. The *localizer* experiment involved a visually cued finger movement. A single trial comprised 12 s of movement followed by 18 s rest. The experiment comprised 10 trials; during even numbered trials the subject moved their left index finger and during odd numbered trials the subjects moved their right index finger allowing both the right and left motor cortices to be identified. Echo planar images (matrix size 144 × 144, TE = 25 ms, SENSE factor = 3) were acquired with a voxel size of 1.5 mm × 1.5 mm × 3 mm. The TR was 2 s for the localizer, but reduced to 1.5 s for the resting state experiment to increase temporal degrees of freedom and therefore improve characterization of temporal correlation. To ensure a homogeneous B_0_, a parcellated shimming procedure (Poole and Bowtell, 2008) was employed. The flip angle was set to the Ernst angle (70°). 24 axial slices were acquired with whole brain coverage. During all experiments the respiration and vector-cardiogram were recorded. Subject head motion was measured during post processing and if any subject moved more than the smallest voxel dimension (1.5 mm) during the experiment, data from that subject were discarded.

### Data analysis

#### Analysis of localizer data

fMRI localizer data were motion corrected (SPM5), corrected for physiological artifact using RETROICOR (Glover et al., 2000), and smoothed spatially using a 3 mm FWHM Gaussian kernel (SPM5). In order to identify areas exhibiting significant BOLD change during finger movement, data were processed using the general linear model implemented in SPM5 (http://www.fil.ion.ucl.ac.uk/spm/). Robust statistically significant (p < 0.05 FWE corrected) activation was observed in all subjects in the left and right sensorimotor cortices. The peak in BOLD activity in the right sensorimotor cortex was used to define a seed location for subsequent fcMRI analyses. The 7T EPI localizer data and associated functional images were co-registered to the 3T anatomical MPRAGE image of each individual subject using the fMRIB linear image registration tool (FLIRT) in FSL (http://www.fmrib.ox.ac.uk/fsl/). Functional images were then co-registered to the standard brain (i.e. MNI space), using FLIRT.

Synthetic aperture magnetometry (Robinson and Vrba, 1998) was applied to the MEG *localizer* data in order to localize left and right sensorimotor cortices. An ‘active’ time window was chosen as the period during which subjects moved both their left and right index fingers. A ‘control’ window was defined as the rest period for each trial. Pseudo-T-statistical functional images were constructed by contrasting beamformer projected oscillatory power in the active window with that in the control window (regularization parameter μ *= 4*; for definition of μ, see below). This resulted in a volumetric image showing the spatial distribution of oscillatory power change. In agreement with previous literature (see e.g. (Stevenson et al., 2011) robust task induced loss in β (13–30 Hz) band oscillatory power was found localized to primary sensorimotor cortices. These locations were later used to define locations of interest in the left and right sensorimotor cortices for MEG FC analysis. MEG localizer images were co-registered to the MNI brain using FLIRT.

#### Analysis of resting state fcMRI data

Resting state fMRI data were motion corrected (SPM5), corrected for non-neuronal physiological artifact using RETROICOR (Glover et al., 2000), and smoothed spatially (SPM5) using a 3 mm FWHM Gaussian kernel. A seed based correlation technique (as described in Biswal et al., 1995) was employed to assess resting state connectivity. An average BOLD seed timecourse was derived from the peak voxel in right sensorimotor cortex, defined from the localizer experiment, and its 26 nearest neighbors. The Pearson correlation coefficients between the seed timecourse, and all voxels in the brain were computed resulting in a volumetric image of correlation coefficients. Correlation coefficient images were thresholded at a value corresponding to p < 0.05 Bonferroni corrected for multiple comparisons (Hale et al., 2010). Again, the 7T EPI data and BOLD FC images were co-registered to the 3T anatomical MPRAGE image of each individual subject, and to the standard MNI brain, using FLIRT.

#### Analysis of resting state MEG data

MEG data were mean corrected and frequency filtered into seven bands of interest using a finite impulse response filter in NUTMEG (http://nutmeg.berkeley.edu) (Dalal et al., 2004). The frequency bands used were δ (1 Hz–4 Hz), θ (4 Hz–8 Hz), α (8 Hz–13 Hz), low β (13 Hz–20 Hz), high β (20 Hz–30 Hz), low γ (30 Hz–40 Hz) and high γ (40 Hz–70 Hz). FC was measured using a three step process: A) application of spatial filters to project data into source space; B) application of the FC metric; and C) weights correlation computation and simulation to assess the statistical significance of the FC metric used. These three steps are described in detail in the following three sections.

A) Application of spatial filters

Frequency filtered MEG data were projected into source space using a scalar beamformer. The electrical source amplitude, ${\hat{Q}}_{\theta }\left(t\right)$ at a predetermined location was estimated as:

(1) $${\hat{Q}}_{\theta }\left(t\right)=W_{\theta }^{\text{T}}m\left(t\right)$$

where **m**(*t*) is the vector of magnetic field measurements made at *M* MEG sensors at time *t*. W_θ_ is a vector of weighting parameters tuned to location and orientation **θ** = [**r**, *δ*], where **r** represents location, and *δ* is the angle of the source with respect to the azimuthal direction. **W**_**θ**_ was computed based on minimization of output signal variance, with a linear constraint that signal power originating at **θ** remains in the output signal (Van Drongelen et al., 1996; Van Veen et al., 1997; Robinson and Vrba, 1998), mathematically:

(2) $$W_{\theta }^{\text{T}}={\left[L_{\theta }^{\text{T}}{\left[C+\mu \sigma I\right]}^{-1}L_{\theta }\right]}^{-1}L_{\theta }^{\text{T}}{\left[C+\mu \sigma I\right]}^{-1}$$

where **L**_**θ**_ represents the lead fields, **C** represents the *M* × *M* data covariance matrix, μ is the matrix regularization parameter, σ is an estimate of uncorrelated noise power and **I** is the identity matrix. The lead fields **L**_**θ**_ were computed using a multi-sphere head model (Huang et al., 1999) and the forward calculation described by Sarvas (1987). The angle *δ* was found independently at each **r** using a non-linear search to compute the orientation of maximal variance. The noise estimate (σ) was computed as the minimum singular value of the covariance matrix and μ = 4 in all cases. The channel level data covariance matrix **C** was computed in two ways: in the first case, only those data acquired during the resting state phase of the experiment were used. In the second case, data from the entire experiment were used. It is well known that the spatial resolution of beamformer imaging is inhomogeneous across the head, with the highest spatial resolution being in regions of high power (Barnes and Hillebrand, 2003). During the resting state phase, brain areas may exhibit little power and undergo no modulation, meaning that spatial resolution may be low and homogeneous. However, using covariance computed over the entire experiment, spatial resolution would be expected to improve, partly due to increased amount of data (Brookes et al., 2008a,b), but also particularly in the motor network which will undergo power change due to the localizer task. This implies that covariance computed over longer windows is advantageous; however in doing this one necessarily assumes that sources of interest are stationary throughout the whole experiment. Beamformer weights **W**_**θ**_^*T*^ and associated timecourses ${\hat{Q}}_{\theta }\left(t\right)$ were computed for locations placed at the vertices of a regular 5 mm grid spanning the entire brain. A separate set of beamformer weights was computed for each frequency band in order to optimize beamformer sensitivity to effects of interest in that band (Brookes et al., 2008a,b, 2009, 2010).

B) Resting state functional connectivity metrics

Having projected MEG data into the brain, the second step involves application of FC metrics. In the following description, the beamformer weights (and therefore estimation of δ for each voxel) were computed from the entire dataset (thus allowing optimal spatial resolution). However, in all cases FC metrics were *only* applied to data acquired during the resting phase (i.e. first 300 s) of the MEG experiment, meaning that *we only compute resting state connectivity between brain regions*. For all computations, a seed voxel was chosen in the right sensorimotor area, as defined individually for each subject using the MEG localizer results.

Four separate FC metrics were applied; two based on envelope correlation—termed Average Envelope Correlation (AEC) and Correlation of Averaged Envelopes (CAE); and two based on coherence termed coherence (Coh) and Imaginary Coherence (ICoh). In all cases, connectivity was measured between the projected signal at the *seed* voxel, and that from all other (*test*) voxels in the brain. The four connectivity metrics were computed as follows:

1. AEC: The timecourses of bandpass filtered electrical activity at the seed and test voxels were Hilbert transformed to obtain the analytic signal. The absolute value of the analytic signal was then determined in order to give the envelope of oscillatory power in the frequency band of interest. This we term the *Hilbert envelope* and this technique has been used extensively in previous MEG studies (for a mathematical description see Brookes et al., 2004). The Hilbert envelopes for the seed and test voxels were divided into *n* time segments of equal length Δ. The Pearson correlation coefficient between seed and test Hilbert envelopes was computed within each segment. This gave *n* correlation values, one for each time segment. These were then averaged across segments yielding a single average value which we term *Averaged Envelope Correlation (AEC)*.
2. CAE: The timecourses of electrical activity at both the seed and test voxels were Hilbert transformed and the *Hilbert envelopes* computed. The Hilbert envelopes were divided into *n* time segments of equal length Δ and the average value of the envelope computed within each time window. This resulted in two new temporally averaged Hilbert envelope timecourses, each comprising *n* points (we term these the ‘*averaged envelopes*’). The Pearson correlation between these averaged envelopes was then computed as a measure of FC which we term *Correlation of Average Envelopes (CAE).*
3. Coh: The filtered projected electrical timecourses (*not the Hilbert envelopes*) from the seed and test voxels were divided into *n* time segments of equal length Δ. Coherence between seed and test timecourses was computed within each segment giving *n* values, one for each time segment. These were then averaged across segments yielding an average *coherence* value *(Coh)*. (Coherence within a single segment, *k*, was computed as $C_{\mathrm{XY}}(f)=\left|\left(X_{k}(f)Y_{k}*(f)\right)/\sqrt{{\left|X_{k}(f)\right|}^{2}{\left|Y_{k}(f)\right|}^{2}}\right|$ for a single frequency bin (*f*) where *X*_*k*_(*f*) and *Y*_*k*_(*f*) are the Fourier transformed segment (*k*) of the virtual time series and * indicates the complex conjugate. Coherence values for frequency bands were computed from the sum of autospectra and cross spectra in the corresponding frequency bins (Guggisberg et al., 2008)).
4. ICoh: As above, the projected electrical timecourses (*not the Hilbert envelopes*) from the seed and test voxels were divided into *n* time segments of equal length Δ*.* The imaginary part of coherence between seed and test timecourses was computed within each segment. The absolute value of imaginary coherence was then computed and averaged across segments *(ICoh)*. (Imaginary Coherence within a single segment was computed as IXY(f)=ImXk(f)Yk*(f)/Xk(f)2Yk(f)2 and values for frequency bands were computed from the sum of autospectra and cross spectra in the corresponding frequency bins (Guggisberg et al., 2008). The absolute value of imaginary coherence was computed since we were interested in the magnitude of FC, not the directionality).

As mentioned above, previous work (Singh et al., 2002; Brookes et al., 2004; Winterer et al., 2007; Stevenson et al., 2011; Zumer et al., 2010) has shown a close relationship between task driven fluctuations in oscillatory power and the hemodynamic response. Further, papers investigating MEG FC and its relationship to fcMRI (de Pasquale et al., 2009; Liu et al., 2010) have both employed amplitude envelope based measurements. This motivated the use of the AEC methodology described above. CAE employs MEG time series that are temporally collapsed. This makes correlation results less sensitive to phase jitter in the Hilbert envelope, and to noise. The CAE measurement is also similar to that used by de Pasquale et al. (2009) however it does sacrifice some temporal resolution. Coherence and imaginary coherence have been applied in previous MEG studies. Here they are employed as a comparison to amplitude envelope based measurements, and to investigate the nature of electrodynamic connectivity. For example, strong agreement between Coh and AEC might imply that phase locking between large cell populations was underlying changes in oscillatory amplitude and driving amplitude based FC measurements. High ICoh values would show that an observable phase lag was apparent between network nodes. Variation of Δ and computation of FC timecourses enable important information about the time scale FC to be generated. Use of the metrics in this way was therefore expected to exploit the direct nature of MEG and provide information on the electrical processes mediating FC.

C) Weights correlation, simulation and statistical significance

Source reconstruction of MEG data limits the effect of field spread since it enables visualization of FC in source space. However, the inverse problem is ill posed and the estimated timecourses of electrical activity at separate MEG voxels are not necessarily independent. In other words, it is possible to get signal leakage between spatial filters. Independence of two spatial filters can be assessed by examining correlation between beamformer weights. If the weights for two spatially separate voxels are correlated (as is likely to be the case for nearby voxels) then the projected signals will also be correlated and this may appear as spurious FC in the beamformer based FC images. If, however, the beamformer weights for two voxels are completely independent, but the timecourses from those voxels are highly correlated, it is more likely that genuine FC exists between those two brain locations. In order to assess the weights correlation problem, correlation coefficients between beamformer weights at the seed location, and all other voxels in the brain were computed in order to create volumetric images of weights correlation. These were constructed for all subjects and all frequency bands (note, the absolute value of weights correlation was computed in all cases). Correlation between beamformer weights was compared to correlation between lead field patterns in order to assess the advantages of beamforming over non-adaptive algorithms (e.g. dipole fitting) in which lead fields are used directly for source reconstruction. Both weights correlation and lead field correlation were plotted against distance between the seed and test voxel in order to examine the extent of spurious connectivity around the seed region.

While weights correlation is a useful metric for visualization of signal leakage, it cannot be used to assess directly the statistical significance of FC and for this purpose, simulated MEG data were generated. On each iteration of the simulation, two dipolar sources were simulated in the brain, the first at the *seed* location, and the second at a *test* location. The timecourses for these two sources comprised Gaussian random noise which was colored by frequency filtering to the band of interest. *No significant correlation existed between simulated seed and test timecourses.* The source orientations and the variance of the source timecourses were equivalent to those derived by application of the beamformer to the real MEG data for the same subject, location and frequency band. The simulated timecourses were multiplied by lead fields for the two locations/orientations and 300 s of simulated MEG data were constructed (taking into account the third order gradiometer sensor configuration). 300 s of MEG data were acquired (using the third order gradiometer configuration of the 275 channel system at a sampling rate of 600 Hz) with no subject in the scanner. These noise data were added to the simulated data resulting in a simulated MEG dataset.

The simulated data were used repeatedly in order to assess statistical significance of measured FC values throughout this paper. On each repetition of this simulation, different seed and test timecourses were employed. Simulated MEG data were projected into the brain using the same beamformer weights derived from and applied to the real MEG data. Since our simulated data were designed to be similar to the real resting state data, beamformer reconstruction of the simulated source timecourses was successful. However no correlation between simulated sources was introduced, meaning that following beamformer projection, if FC analysis of simulated data implied high levels of FC between sources, this was entirely spurious and could be due to either weights correlation, field spread between the two sources or real correlated noise across MEG sensors.

Two separate strategies were employed to identify areas of statistically significant FC in MEG images; the first technique was applied to individual subject data and the second to group data. In both cases, volumetric images containing simulated FC values were employed and for every FC image created using real MEG data, an equivalent simulated FC image was created. (Note: in creating the simulated FC image, on each iteration of the simulation, the test voxel was moved to a different location, meaning that a single MEG dataset was simulated for every voxel.)

In order to derive statistical significance in a single subject the following process was employed: 1) voxels were binned according to their weights correlation (bin width 0.05) and the mean value of simulated *FC* was computed within each bin; 2) The mean simulated FC values within each bin were subtracted from values derived from real and simulated data on a voxel by voxel basis—the result being two corrected images, one based on real data, the other on simulated data; and 3) The distributions of corrected simulated *FC* values across all voxels were computed and used to threshold corrected *FC* images derived from real data. Only those voxels in the upper fifth percentile of the corrected simulated distribution were said to exhibit statistically significant FC.

In order to assess statistical significance of FC across the subject group, FC images were co-registered to the standard brain using FLIRT. Following co-registration, in a single frequency band, for every voxel in MNI space, six real and six simulated FC values were available (one value per subject). These two sets of numbers were averaged, and the statistical significance of the difference between them was computed using a non-parametric Wilcoxon signed rank test, implemented in Matlab (Mathworks). Under a null hypothesis, one would expect no significant difference between the real and the simulated data. Voxels in which a statistically significant (p < 0.05) difference between the real and the simulated images was observed were said to exhibit significant FC at the group level.

#### Investigating electrodynamic connectivity

FC between the left and right motor cortices is a well known effect in fcMRI. For this reason, investigation into the nature of inter-hemispheric connectivity was undertaken by measurement of FC between 2 locations; a seed in the right motor cortex and a test voxel in the left motor cortex. Both locations were defined independently for each subject based on the results of the MEG localizer analysis. Beamformer spatial filters were derived for both locations and resting state data were projected into the brain. *AEC*, *CAE*, *Coh* and *ICoh* were computed between locations in left and right motor cortex (as described above). This was done independently for all frequency bands and subjects; Δ was varied, taking values of 0.5 s, 1 s,4 s, 6 s and 10 s.

To test the significance of FC between the two locations, 100 simulated MEG datasets were constructed (as described above) with simulated seed and test sources in the right and left motor cortices respectively. (Again this was done for each frequency band and subject, with source orientation and amplitudes matched to those measured from real data.) Simulated datasets were projected through the same spatial filters as those used for real data and 100 simulated values of *AEC*, *CAE*, *Coh* and *ICoh* were derived (again for varying Δ). The mean of the simulated values was subtracted from values derived from real data on a subject by subject basis yielding *corrected AEC*, *CAE*, *Coh* and *ICoh* values. These corrected values were then averaged across subjects and standard error computed. Finally, the simulated values were mean corrected (on a subject by subject basis) and concatenated (across subjects) to create a distribution comprising 600 points for each of the four connectivity measures and each of the seven frequency bands. The 95% confidence limit was computed and used to assess statistical significance of the measured FC. Using the null hypothesis, one would expect no significant difference between *AEC*, *CAE*, *Coh* and *ICoh* values derived using real and simulated data.

Previous results (de Pasquale et al., 2009) have shown that the time at which FC is measured can have an important effect on the results obtained. In their paper de Pasquale et al. show that cross hemisphere FC was better observed in carefully selected short time windows than it was using their entire MEG dataset. In order to assess this effect in our study, the timecourses of FC between left and right sensorimotor cortices were assessed. As described above, *AEC*, *Coh* and *ICoh* were computed within *n* time windows of length Δ. This enabled visualization of the timecourse of FC, with temporal resolution determined by Δ. The *AEC*, *Coh* and *ICoh* timecourses were plotted in order to gage the variation of the FC metric. In addition, FC timecourses enabled an interesting comparison between our metrics since agreement (or disagreement) between metrics would provide valuable information about the relationship between, for example, envelope correlation and coherence. For this reason, the correlation between FC timecourses measured using *AEC*, *Coh* and *ICoh* was computed allowing a numerical measure of the similarity between metrics and enabling us to probe the relationship them.

#### Cross modal comparison

A comparison between fcMRI and fcMEG was undertaken by assessment of the similarity of FC images derived using the two modalities. Initially, a visual comparison was made between FC images generated using fcMRI and MEG. The locations of statistically significant correlated areas were recorded, and the similarity between peak locations measured. This was undertaken for each of the 7 frequency bands used in MEG analysis and allowed a visual comparison of those MEG frequency bands most closely related to FC measured using fcMRI.

In order to obtain a numerical value for the spatial similarity between MEG and fcMRI, a spatial correlation coefficient metric was introduced. Initially, the fcMRI maps (in MNI space) were smoothed using a Gaussian smoothing kernel (FWHM 8 mm) (this was to account for the intrinsic differences in image smoothness between MEG and fcMRI measurements) and averaged across subjects. The map was then reshaped into a one dimensional vector. Unthresholded MEG FC maps (also in MNI space) were reshaped into one dimensional vectors and the Pearson correlation coefficients between fcMRI and MEG derived vectors were computed to give a numerical measure of the spatial similarity between maps. This metric, which we term *spatial correlation*, was calculated for all frequency bands on a subject by subject basis. Values were averaged across subjects and plotted as a function of frequency. The motivation for this measurement was to see which MEG frequency band best represented the spatial signature of BOLD connectivity. In order to assess significance, the same spatial correlation metric was repeated using FC images derived using simulated MEG data and statistical significance between spatial correlation values computed using real and simulated data was assessed

Finally, the spatial overlap of significantly connected regions, derived from thresholded fcMRI and fcMEG images was computed as a function of frequency.

## Results

The Results section is split into 5 parts. In the first part, we investigate spatial correspondence between fMRI and MEG localizers. In the second part, we address the confounds of MEG FC measurement due to signal leakage between voxels in source space. In the third part we use MEG data to investigate electromagnetic FC between the left and the right sensorimotor cortices. In the fourth part, we undertake a quantitative spatial comparison between fcMRI and fcMEG. In the final part, we address limitations of our simulation approach to eliminating spurious FC.

### Localizer analysis

**Fig. 1:**
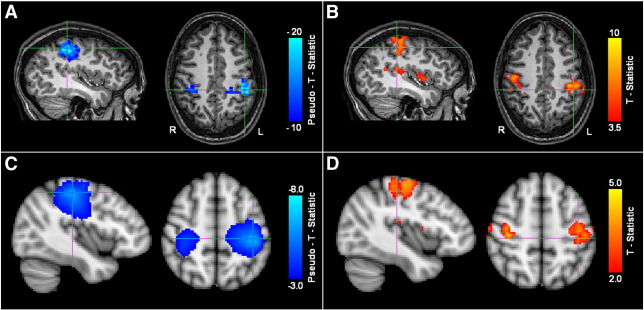
Results of the MEG and fMRI localizer experiments. A) β band power decrease in bilateral motor cortex in response to the finger movement task (single subject); B) corresponding increase in BOLD contrast in bilateral motor cortex (single subject). C) β band power decrease averaged across subjects and overlaid onto the MNI brain; D) corresponding increase in BOLD also averaged across subjects. All images are shown according to the radiological convention. Cross hairs are placed at the peak β band power decrease.

Fig. 1 shows the results from the MEG and fMRI localizer analyses. Fig. 1A shows decrease in β band power in the left and right motor cortices in response to the finger movement task, for a single representative subject. Fig. 1B shows the corresponding increase in BOLD signal, again in a single subject, which also highlights left and right sensorimotor cortices. Figs. 1C and D show equivalent results averaged across the subject group; panel C shows β band power decrease while panel D shows corresponding BOLD increase.

Note that good spatial agreement is observed between β band power loss and increased BOLD signal. The average MNI coordinates for the peaks in β band power loss were (− 41 ± 3, − 25 ± 6, 49 ± 9) mm (left motor cortex) and (34 ± 8, − 31 ± 10, 49 ± 9) mm (right motor cortex). The average locations of the peaks in BOLD response were (− 41 ± 4, − 21 ± 8, 53 ± 6) mm (left motor cortex) and (42 ± 8, − 19 ± 10, 51 ± 9) mm (right motor cortex). (Note that all measurements are quoted as mean ± standard deviation across subjects.) Given the error bounds quoted, the spatial location of the peak BOLD and β band responses were not significantly different. The mean Euclidean distance between β band and BOLD peaks was 17 ± 7 mm. This level of spatial correspondence is in agreement with previously published work (Brookes et al., 2005; Winterer et al., 2007; Stevenson et al., 2011). Discrepancies could be the result of inaccurate coregistration, inaccuracies in MEG inverse modeling, or they may reflect genuine spatial differences brought about by the fundamental differences between these two disparate measurements. For the connectivity results that follow, seed locations for MEG FC analysis were based on each individual's MEG localizer analyses; seed locations for fcMRI were based on each individual's fMRI localizer analyses. In all cases, seed locations were in the right sensorimotor cortex.

### Spatial filter properties

**Fig. 2:**
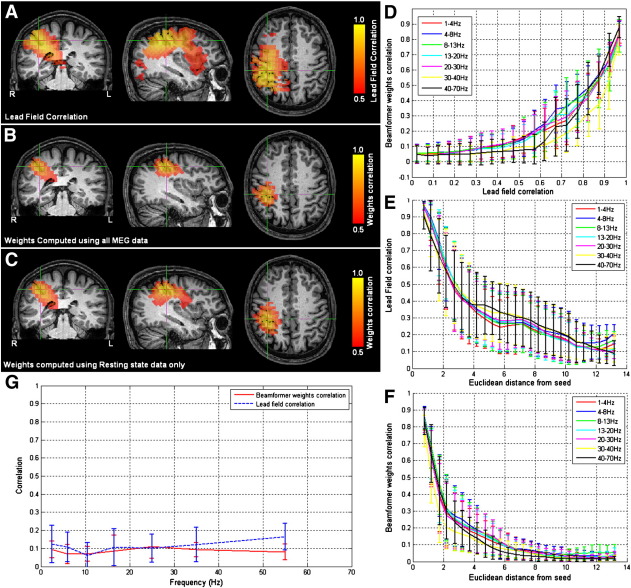
Signal leakage in beamformer spatial filtering: A) Correlation between lead fields at the seed location (cross hairs) and all other brain voxels (result for a single subject). B–C) Volumetric images of correlation between beamformer weights at a seed location (cross hairs) and all other test voxels in the brain. B) Weights computed using contiguous resting state and localizer data (μ = 4; 13 Hz–20 Hz band; single subject); C) weights computed using resting state data only (μ = 4; 13 Hz–20 Hz band; single subject); D) beamformer weights correlation plotted as a function of lead field correlation, highlighting the non-linear relationship between the two metrics (all frequencies; results for a single subject). E) Lead field correlation plotted as a function of distance from the seed location. F) Beamformer weights correlation (weights computed using all data; μ = 4) plotted as a function of distance from the seed location. G) Weights correlation (red) and lead field correlation (blue) between left and right motor cortices (locations identified from localizer analyses (Fig. 1)) plotted as a function of frequency (weights computed using all data; μ = 4; average and standard error across subjects shown; 13 Hz–20 Hz band).

As stated above, beamformer spatial filters, derived for spatially separate brain locations, are not necessarily independent and cross talk between voxels could cause spurious FC measurement. It is therefore important to determine accurately the properties of spatial filters prior to FC computation. Fig. 2A shows a volumetric image of Pearson correlation between lead fields at the seed location (at the cross hairs) and lead fields at all other source space voxels. This is shown for a single subject, with the source orientation for each voxel derived as the orientation of maximum signal variance. Pearson correlation coefficients are thresholded at 0.5 for visualization. Figs. 2B and C show volumetric images of correlation between beamformer weights at the seed location and at all other voxels in the brain. (Weights correlation images are computed using 13 Hz–20 Hz filtered MEG data.) Fig. 2B shows weights correlation in a case where covariance is based on all available MEG data (i.e. contiguous resting state and localizer data); Fig. 2C shows weights correlation in a case where covariance is based on resting state data only. Images are thresholded at 0.5. Notice here that weights correlation is far less widespread than lead field correlation, an effect of the adaptive nature of beamforming which shows that, even if lead fields are correlated, beamformer weights can remain independent.

Demarcation between lead field and weights correlation is confirmed in Fig. 2D which highlights (for all frequency bands) the non-linear relationship between the two. Again, this shows that high correlation between lead fields does not necessarily imply high correlation between beamformer weights. This effect is affected by signal power; the beamformer is able to separate two voxels with overlapping lead fields only if significant signal power originates at those two voxels. This has been addressed in previous work (Barnes and Hillebrand, 2003; Brookes et al., 2008a,b, 2009, 2010). It is important to note that using covariance based on all data (i.e. resting state and localizer data), the total volume of cortex with weights correlated at *r* > 0.25 was 126 ± 15 cm^3^; this was compared to 155 ± 17 cm^3^ when weights were computed using resting state data only (results given as average and standard error across subjects). This highlights the advantage of judicious selection of a time frequency window for weights computation and shows that inclusion of data recorded during a task driven change in signal power can improve the spatial resolution of the beamformer. Figs. 2E and F show lead field correlation and weights correlation as a function of distance from the seed location respectively. Note the improved spatial resolution of weights correlation with respect to lead field correlation that is also apparent in Figs. 2A and B. (Note also that separate lead field correlations for different frequency bands appear because the source orientation (δ) is computed independently for each frequency band. Lead fields themselves do not change with frequency.)

The ability of the beamformer to construct independent weighting parameters, even in the case of correlated lead fields, makes it advantageous compared to non-adaptive source localization algorithms which rely only on lead fields to reconstruct source space signals. This is true of all beamformer applications, but is particularly important for FC measurement since high values of FC will necessarily result from correlation between weights. Here, we are interested specifically in connectivity between the left and right motor cortices and, in all subjects, weights correlation did not extend from the seed in the right motor cortex to the left motor cortex. This is shown in Fig. 2G where weights correlation between locations of interest in left and right sensorimotor cortices is plotted as a function of frequency (average ± standard error across subjects). Notice that correlation between left and right sensorimotor cortices is low (~ 0.1) and shows no significant change across frequency bands. The graph also shows lead field correlation between the left and right motor cortices which also remains low for all frequencies. (Note again that the small variation in lead field correlation across frequencies is due to the slight difference in the estimation of δ for the frequency bands studied.)

### Investigating electrodynamic connectivity

The results above show that beamforming is an effective source localization algorithm and further that since weights derived for the left and right sensorimotor cortices are independent, measurement of high FC metrics is likely to result in real, not spurious FC measurement. In this section we present the results of our investigation into electrodynamic connectivity between the left and right motor cortices. We exploit the direct nature of MEG, and the multiple FC metrics described, to investigate the electrodynamic processes that underlie FC.

**Fig. 3:**
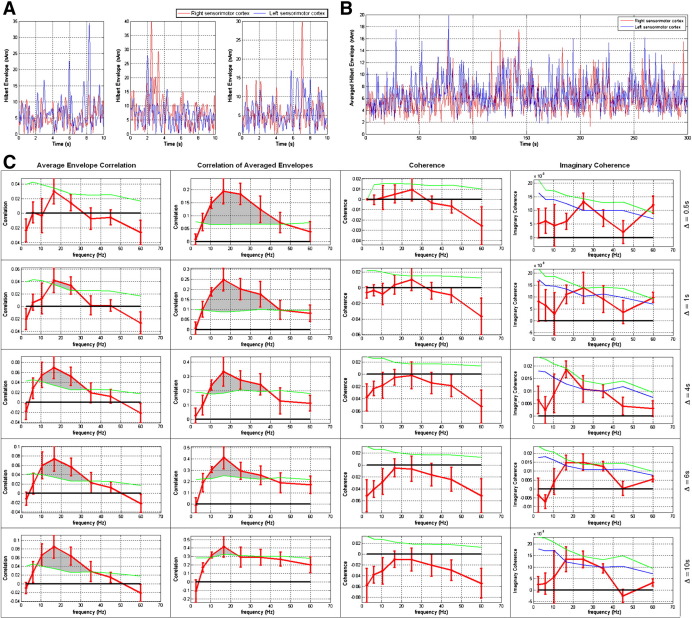
Cross hemisphere connectivity measurement: A) Hilbert envelope timecourses extracted from the left (blue) and right (red) sensorimotor cortices of a single subject. The three panels show three separate (Δ = 10 s) segments that are used (alongside 27 others) in an averaged envelope correlation computation. B) Averaged Hilbert envelope timecourses (Δ = 0.5 s) extracted from the left (blue) and right (red) sensorimotor cortex of a single subject. These two timecourses are used to obtain the correlation of averaged enveloped (CAE) metric. C) Corrected AEC (Column 1), CAE (Column 2) Coh (Column 3) and ICoh (Column 4) values, plotted as a function of frequency. In all cases the red line shows the result from real data (average and standard error across subjects) while the green line shows the 95% confidence limit derived from simulations. In the case of ICoh, the blue line shows the 90% confidence limit. The five rows in Fig. 3C show results for Δ = 0.5 s, 1 s, 4 s, 6 s and 10 s respectively. Gray shading indicates statistical significance (p < 0.05) across the subject group.

Fig. 3A shows an example of the Hilbert envelopes derived from locations of interest in the left (blue) and right (red) sensorimotor cortices. These timecourses were taken from a single subject; the locations were derived based on that subject's localizer analysis and the three panels show three separate 10 s segments of data. Fig. 3B shows an example of the averaged Hilbert envelope timecourses (Δ = 0.5 s) extracted from the left (blue) and right (red) sensorimotor cortex. Again this result is derived from a single subject and all 300 s of resting state data are shown. In both Figs. 3A and B, data have been filtered to the low β frequency band.

Fig. 3C shows corrected *AEC*, *CAE*, *Coh* and *ICoh*, applied to signals extracted from the left and right motor cortices. Note in all cases that the locations of interest were derived individually for each subject based on the localizer analysis. The left hand column shows corrected *AEC* between the left and right motor cortices, plotted as a function of frequency. To derive the corrected value, the average *AEC* from simulated data was subtracted from that derived from real data, on a subject by subject basis. (In order to improve visualization of the characteristic frequency response, for this analysis, the high-γ frequency band (40–70 Hz) was split into two, 40–50 Hz and 50–70 Hz.) A threshold was derived using the statistical distribution of AEC values from simulated data (see Methods section) and corrected *AEC* values above a threshold corresponding to p = 0.05 were taken to be significant. (These regions are shaded in gray.) The 5 rows of Fig. 3C show the case for Δ = 0.5 s, 1 s, 4 s, 6 s and 10 s respectively. The second column of Fig. 3C shows corrected *CAE* between the left and right motor cortices as a function of frequency (again Δ = 0.5 s, 1 s, 4 s, 6 s and 10 s are shown and correction is performed in the same manner as for *AEC*). The third column shows corrected *Coh* values between the left and right motor cortices and the final column shows corrected *ICoh* values. In all cases the red line shows the result (average and standard error across subjects) while the green line the 95% confidence limit based on simulations. In the case of ICoh, the blue line shows the 90% confidence limit, based on simulation. Raw values of AEC, CAE, Coh and ICoh, applied to real and simulated MEG data, are shown in Appendix 4.

Results show that the group average *AEC* values derived from real data (Δ = 1 s, 4 s, 6 s and 10 s) are significantly larger than those derived from the simulated data, implying significant connectivity between the left and right motor cortices. The group average *CAE* values derived from real data (all Δ) also exhibit statistical significance. For both *AEC* and CAE, a clear frequency band response is observed with the highest FC metrics observed in the low β band (13–20 Hz). This is not surprising since oscillatory effects in the β band are well known to play a fundamental role in the motor network and these effects have been previously reported (Mantini et al., 2007; Liu et al., 2010). Interestingly, the size of the effect observed depends on the time scale (Δ) on which the measurements of correlation are made. This effect provides information on the time scale of functional connectivity and this is addressed in more detail in the discussion below. The *Coh* measurements showed no significant effect in any frequency band. *ICoh* values were extremely small in magnitude, and did not reach significance (p = 0.05). However, the frequency signature of *ICoh* mirrored that shown by the *AEC* and *CAE* metrics with the highest *ICoh* being observed in the β band. In addition (for Δ = 4 s), *ICoh* values exceeded a 90% confidence limit.

**Fig. 4:**
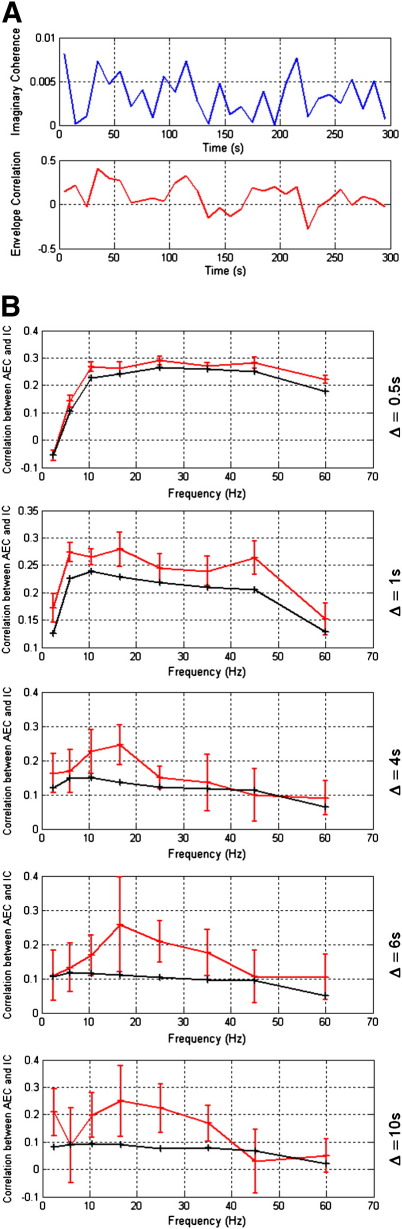
Investigating a relationship between envelope correlation and phase coupling. A) The timecourse of FC measured using ICoh (upper panel), and AEC (lower panel) for (Δ = 10 s). B) Correlation between ICoh and AEC derived FC timecourses for Δ = 1 s, 4 s, 6 s and 10 s. Red line shows result derived using real MEG data while black line shows result from the simulations.

The results shown in Fig. 3C represent FC averaged over a 300 s window. However, recent interest has grown in dynamic FC measurements (Chang and Glover, 2010). Fig. 4 shows that dynamic measurements of electrodynamic FC can be obtained using our ICoh and AEC techniques. For both metrics, *n* FC measurements are made using *n* time windows, enabling a timecourse of FC to be derived whose temporal scale is determined by Δ. Fig. 4A shows two such timecourses, the upper panel shows *ICoh* while the lower panel shows *AEC* (Δ = 10 s in both cases and 13 Hz–20 Hz filtered data are used). Notice that the *ICoh* values, peaking at around 0.07, are much smaller than the *AEC* values which peak around 0.4. Notice also that there is a large variation in FC over time, with AEC values ranging from 0 to 0.4. Fig. 4B shows correlation between *ICoh* and *AEC* FC timecourses as a function of frequency for Δ = 1 s, 4 s, 6 s and 10 s. The red line shows the result for real data while the black line shows the result derived from simulated data. Notice that for Δ > 4 s, a peak is observed in the β frequency band which is only apparent for real data. These peaks do not reach statistical significance (p = 0.05 derived using the simulated data) across the subject group. However, the trend observed does imply that, in the β band, a degree of similarity exists between *AEC* and *ICoh* and that coherent activity (with a non-zero time lag) may underlie envelope correlation.

### Cross-modal comparison

**Fig. 5:**
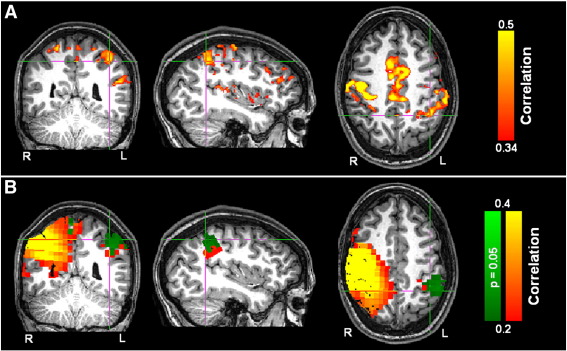
Comparison between fcMRI and fcMEG in a single subject. A) Statistically significant (p < 0.05) correlation between a BOLD timecourse at the seed in the right motor cortex, and all other voxels. B) Correlation between averaged Hilbert envelopes at the seed and all other voxels for MEG data filtered to the low β band (13 Hz–20 Hz). The green overlay shows voxels achieving statistical significance (p < 0.05). Note good agreement between the location of the peak in the left motor cortex for fcMRI and fcMEG. All images are shown according to the radiological convention.

A comparison between resting state FC measured using fcMRI and MEG was undertaken based on the similarity between FC images derived from the two modalities. Since our CAE metric yielded the highest temporal correlation coefficients, this metric was used exclusively for the cross-modal comparison. Fig. 5 shows FC images acquired in a single subject. Fig. 5A shows the fcMRI result, with a seed in the right motor cortex yielding significantly (p < 0.05) correlated voxels in left primary sensorimotor cortices and supplementary motor area (SMA). The strongest correlation was in left primary sensorimotor cortices. Fig. 5B shows an equivalent result derived using MEG. Here CAE (Δ = 0.5 s) is applied to low β band filtered MEG data. As with fMRI, a seed in right sensorimotor cortex yields significant correlation with voxels in the left primary sensorimotor area (p < 0.05). In Fig. 5B, the red–yellow overlay shows raw correlation values while the green overlay shows those correlation coefficients that are significantly higher than equivalent correlation values computed in simulation. Note that the highly correlated diffuse region around the seed in right sensorimotor cortex is a result of signal leakage between voxels and can be explained by weights correlation; the region in left sensorimotor cortex cannot be explained by leakage and therefore represents genuine connectivity. Given the fact that no a-priori fcMRI based information was used in the MEG source reconstruction, or connectivity analyses, the level of spatial agreement between these images is compelling.

A high level of spatial agreement between FC images was observed across the subject group in both the low β and the high β MEG frequency bands. Table 1 shows the MNI coordinates of the highest peaks in the left sensorimotor area derived from fcMRI and fcMEG images. Note that since the best spatial agreement between fcMRI and fcMEG was observed in the β band, results are shown for the 13–20 Hz and the 20–30 Hz bands only. These results are averaged across subjects and mean ± standard deviation is reported. The localizer results are also shown for comparison.

**Fig. 6:**
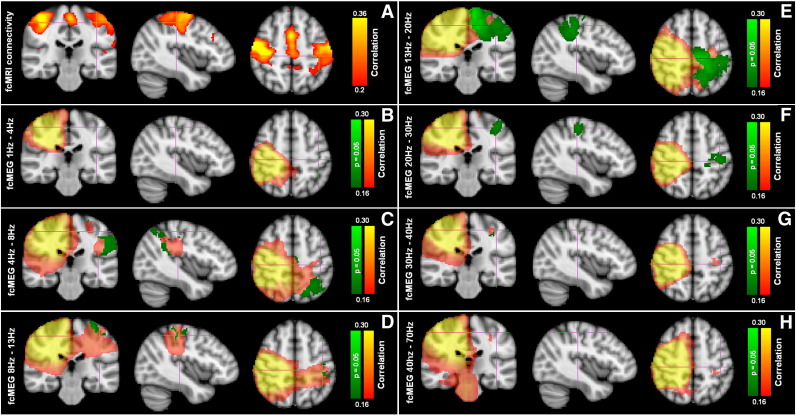
Group results. A) Functional connectivity MRI results averaged across 5 subjects. B)–H) Correlation of averaged envelope results in the 1 Hz–4 Hz, 4 Hz–8 Hz, 8 Hz–13 Hz, 13 Hz–20 Hz, 20 Hz–30 Hz, 30 Hz–40 Hz and 40 Hz–70 Hz frequency bands respectively. The green overlay shows voxels in the left hemisphere exhibiting statistically significant (p = 0.05) correlation across the subject group. Notice that in the β band there is good spatial agreement between fcMEG and fcMRI results. All images are shown according to the radiological convention.

The spatial agreement, highlighted in Table 1, is also shown in Fig. 6. Fig. 6A shows the group average FC map derived from fMRI (thresholded at a correlation coefficient of 0.2). Figs. 6B–H show CAE analysis (Δ = 0.5 s) applied to MEG data for all frequency bands: B: 1 Hz–4 Hz; C: 4 Hz–8 Hz; D: 8 Hz–13 Hz; E: 13 Hz–20 Hz; F: 20 Hz–30 Hz; G: 30 Hz–40 Hz; and H: 40 Hz–70 Hz. In all fcMEG images, the green overlay shows voxels in the left hemisphere exhibiting statistically significant (p = 0.05) correlation with the seed in the right hemisphere. Note that significance is assessed across the subject group employing simulated data and a non-parametric Wilcoxon signed rank test. For all of the images shown, the cross hairs are at the same location in MNI space (− 40, − 26, and 52) mm. Functional images are not corrected for multiple comparisons. Significant left hemisphere connectivity is observed in the θ, α, β and low γ bands, however the best spatial agreement between fcMRI and fcMEG is seen in the β band, with the most focal region of high correlation observed in the high β (20–30 Hz) frequency band. Note that connectivity in the θ band is spatially distinct and does not overlap with that observed using fcMRI.

**Fig. 7:**
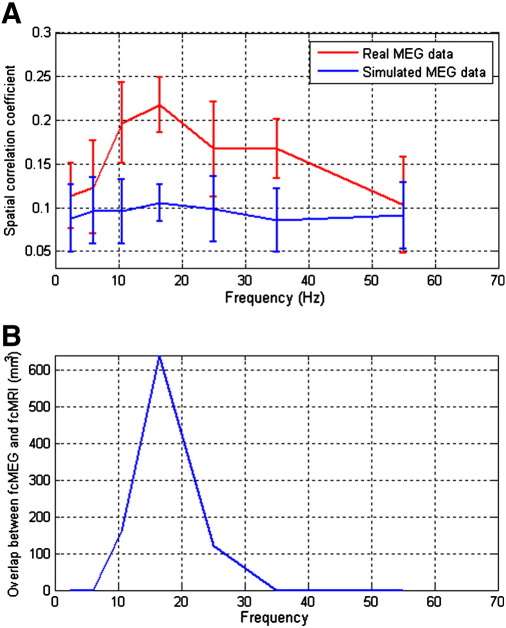
Quantitative comparison of fcMRI and fcMEG images. A) Spatial correlation coefficients between unthresholded fcMEG images and the unthresholded average fcMRI image as a function of frequency. Red line shows real MEG data while the blue line shows the case for fcMEG images derived using simulated data. A significant (p < 0.05) difference between these two measurements occurs in the low β frequency band. B) Spatial overlap between significant connectivity in left hemisphere identified using fcMRI and fcMEG. Note in both cases a peak in the β band.

Fig. 7 shows results of a quantitative comparison of the similarity between fcMEG and fcMRI images. Fig. 7A shows the spatial correlation coefficient derived between the unthresholded fcMEG images and the unthresholded average fcMRI image for each frequency band studied. The red line shows spatial correlation between CAE images derived from real MEG data and fcMRI, while the blue line shows spatial correlation between CAE images derived using simulated data and fcMRI. Here, spatial correlation has been computed on a subject by subject basis and the result averaged across subjects. Note that in the low β band, images derived from real data exhibit significantly (p = 0.0312) higher spatial correlation than those derived using simulated data. In addition, spatial correlation between fcMEG and fcMRI derived using the 13 Hz–30 Hz band data is significantly (p < 0.05) higher than equivalent spatial correlations derived using the 1 Hz–4 Hz and 40 Hz–70 Hz MEG data. Fig. 7B shows the volumar overlap between significant connectivity in left hemisphere identified using fcMRI and fcMEG with overlap peaking in the low β band. In general, deriving active cortical volumes based on beamformer based images is confounded since beamforming is unable to provide an accurate measure of source extent. Here we use this metric to show that while overlap is observed in the α and β bands, no overlap is observed in δ, θ or γ. These quantitative results are in agreement with the images in Fig. 6, and show that the best agreement between FC images derived using fcMRI and fcMEG occurs when MEG data are frequency filtered into the β band. This point will be addressed further in the discussion.

### Remaining issues

There are 2 key questions that remain to be addressed in regarding the results of this study. Firstly, how does the frequency content of the Hilbert envelope affect FC metrics? Secondly, how does interference from other biomagnetic sources, either in the brain or elsewhere, affect FC metrics? These issues are dealt with in Appendices 1–3; The key results are given here.

A potential limitation of our simulations lies in the generation of the source timecourse data. The white noise data employed for timecourse simulation contains no temporal structure in its envelope. The Hilbert envelopes of the true β band data may therefore contain frequency components that are not contained in the simulated data. As a result, the differences between real and simulated data in Fig. 3C could be interpreted as true bivariate coupling, or alternatively a difference in univariate data statistics. If the latter was the case, then the results presented in Figs. 5, 6 and 7 would suggest that those differences (temporal structure vs. no temporal structure) are not a general cortical phenomenon, but only occur in the motor cortices and occur maximally in the β band, making this interesting in itself, but not truly reflective of FC. This ambiguity can however be lifted by a modification to the simulations whereby the frequency content of the real and simulated Hilbert envelope data is matched. In Appendix 1 we undertake this measurement; we show that when the frequency content of envelope signals is matched, FC values are largely unaffected suggesting that the effects observed in Fig. 3 reflect true bivariate coupling.

Our simulation approach to removing spurious connectivity, while effective and more conservative than other approaches, does not represent a perfect solution to eliminate all spurious connectivity. The simulations account for spurious FC resulting from beamformer weights correlation (i.e. cross talk between spatial filters), field spread between the two sources of interest or external interference correlated across sensors. The simulations do not account for interference from other biomagnetic sources; for example, it is possible that a third source (located in the brain and not accounted for in simulation) may interfere with both the seed and test sources and thus cause spurious connectivity. It is also possible that non brain sources (e.g. cardiac/respiratory sources) may also interfere and lead to spurious FC. We assume that these additional biomagnetic sources are eliminated by the beamformer. Previous work (Sekihara et al., 2004, 2006; Brookes et al., 2008a,b, 2009, 2010) has shown that beamforming represents an effective interference suppression tool and so this assumption is reasonable. In Appendix 2, we apply a simple modification to our simulations to examine the effect of a third brain source located midway between the left and right motor cortices; results show that beamforming does effectively suppress this source and FC results are unaffected. In Appendix 3 we also show that interference due to the cardiac cycle is dramatically reduced by the use of a beamformer spatial filter.

## Discussion

In this paper, we have described methodologies for measurement of resting state functional connectivity in the brain using MEG data. We have shown that it is possible to reconstruct resting state connectivity patterns using MEG that mirror those results obtained using fcMRI. From a MEG methodological point of view, we have shown that beamforming provides an excellent means to measure FC in source space. However, care must be taken when making such measurements since cross talk between voxels can potentially lead to spurious connectivity and this effect must be taken into account in all studies of this type. The spatial agreement between FC measured using MEG and fcMRI has shown that β band effects are implicated in inter-hemispheric motor cortex connectivity. This spatial agreement helps to reduce the potential confounds associated with each modality alone: while it helps reduce the uncertainties in spatial patterns generated by MEG (brought about by the ill posed inverse problem), addition of an electrodynamic metric confirms the neural basis of fcMRI measurements. Finally we have demonstrated the application of multiple MEG FC metrics (*CAE*, *AEC*, *Coh*, and *ICoh*). These methods should not be considered as competing techniques but rather complementary methodologies that outline the potential of MEG as a tool to move beyond hemodynamic responses and gain a better understanding of the electrical nature of brain FC.

### Beamformer methodology and FC measurement

The technique described for MEG based FC measurement is centered on the application of a beamformer spatial filter. The suitability of beamforming for FC measurement has been highlighted in studies involving DICS (Gross et al., 2001). A distinct advantage of beamforming is that it is an adaptive source localization algorithm and signals that do not originate from the brain (i.e. those signals whose spatial profile at the sensor level cannot be explained by a dipolar source inside the head) are suppressed. This gives beamforming an interference rejection property which is not mirrored by non-adaptive algorithms. Interference rejection has been discussed in previous papers (e.g. Brookes et al., 2009, 2010). In Appendix 3 of this paper we highlight a single example showing rejection of cardiac interference. Using channel level analyses, spurious FC measurement could result directly from cardiac interference since the artifact affects a large number of sensors. However, correlation between beamformer projected MEG data and the ECG is small showing that cardiac interference is greatly reduced and so unlikely to affect left–right sensorimotor cortex connectivity. This example represents only one of a number of interference fields; including low frequency respiratory artifacts, the magnetomyogram and environmental noise. All interference fields are actively suppressed by beamforming and this makes it a more attractive artifact rejection technique than alternative methods such as ICA which are subjective since noise components are often manually selected and eliminated.

A major difficulty in measurement of source space FC comes as a result of cross-talk (or signal leakage) between voxels in source space. The ill posed nature of the inverse problem means that voxels are not necessarily independent and as a result, signals from spatially separate brain locations can become correlated even if no genuine connectivity exists. Here, we highlight this by examining both lead field correlation and beamformer weights correlation. If the beamformer weights from spatially separate voxels are correlated, then the reconstructed signals from those voxels will also be correlated (see Eq. (1)) and this will lead to spurious FC. Fig. 2 shows examples of both lead field and weights correlation images. Notice the anisotropic nature of the spatial profile of correlation in both cases; highly correlated regions appear that are distal to the seed location and could easily be mistaken for spatially separate connected brain areas. However, the beamformer weights correlation affects significantly less cortical volume than lead field correlation; this demarcation between lead field and weights correlation is highlighted in the non-linear relationship between the two metrics shown in Fig. 2D. The fact that independent weights can result from correlated lead fields gives beamforming its distinct advantage when compared to non-adaptive algorithms.

Weights correlation can be minimized by judicious paradigm design. Beamformer spatial resolution inhomogeneous, with highest spatial resolution achieved around areas of high signal (Barnes and Hillebrand, 2003). Here weights were constructed using covariance based on 300 s of resting state data and contiguous data recorded during the finger movement paradigm. The increased amount of data, coupled with the high power changes induced in sensorimotor cortices during the finger movement allowed optimal spatial resolution in brain regions identified with the motor network. However, it should be pointed out that even with optimized paradigm design, weights from a significant volume of brain tissue remained correlated with the seed and such effects must be taken into account in future studies (systematic evaluation of nulling beamformer approaches (Dalal et al., 2006) may be of some value here). It also should be appreciated that in constructing weights based on the entire dataset we make the critical assumption that sources in regions of interest are stationary, meaning that source orientation does not change between the resting state and the task. Finally, we should note that while weights correlation has been used here as an informative measure of spatial specificity, this is not the only means to make such measurements. The resolution kernel (see Sekihara et al., 2005 for a complete description) is a means to assess the contribution of distant sources to the estimated activity at a predetermined location. This makes it potentially useful for investigation of signal leakage and hence for future studies addressing new methodology for FC measurement in MEG.

While weights correlation can test the independence of spatial filters, it cannot be used to test directly the validity of FC measurement. It is important to note that weights correlation is certain to result in high FC values, however low correlation between weights does not guarantee complete elimination of spurious FC. With this in mind, in order to test the validity of FC measurement in this paper we employed simulations. In our approach, dipolar sources were simulated at the seed and test locations. These simulated sources had equivalent orientation and amplitude to those measured using the real data; however, their timecourses were random and uncorrelated. Simulated MEG data from these two sources were added to genuine noise data; the resulting sum was projected through the same spatial filters as used to project the real data, yielding beamformer reconstructions of the simulated sources. Connectivity between the seed and test locations was measured using our simulated data. High levels of FC measured using simulated data would be entirely artifactual and a result of either: 1) signal leakage due to correlated beamformer weights; 2) spatial spread of magnetic field induced by current at the two source locations (i.e. the magnetic field from the first source overlapping with sensors used to reconstruct the second source, or vice versa); or 3) correlated MEG noise at the sensor level. All of these effects could result in spurious FC measurement in real MEG data.

**Fig. A7:**
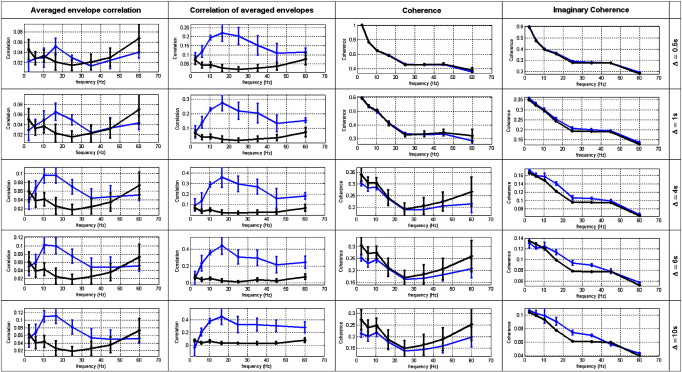
Fig. A7 AEC, CAE, Coh and ICoh metrics applied to real (blue curve) and simulated (black curve) data extracted from left and right motor cortices. The four columns show the four separate FC metrics and the 5 rows show different values of delta.

The accuracy of our simulations is shown to some degree by Fig. A7; notice the good agreement between the real and simulated data in frequency bands, metrics and at time scales (Δ) not exhibiting FC. However, there are potential limitations to our simulation approach. Firstly, simulating data is computationally demanding and while it proved possible for 300 s resting state studies, the approach may be impractical for longer studies. Secondly, since no subject was present during the MEG noise recording, our simulations account for environmental noise, but not for interference from non-neuronal physiology or other brain sources; such effects must therefore be assumed to be attenuated by beamforming (see also Appendix 2). Thirdly, simulated timecourse data were based on white noise meaning that the frequency content of the simulated Hilbert envelope data may differ from that of the real data (see also Appendix 1). There are alternative approaches to testing statistical significance and these usually involve construction of surrogate data directly from the real data. For example, an attractive technique involves randomizing the phase of MEG data acquired at each sensor. This results in a set of surrogate MEG signals with the same power spectrum as the real signals, but randomized phase means that all genuine FC will be destroyed. Projecting these surrogate data through the beamformer spatial filters will result in a methodology for measurement of spurious connectivity. However, this technique, and those that involve switching segments of data in time, necessarily assumes that MEG noise is uncorrelated across sensors. Further the effects of field spread are not taken into account. Therefore, while this approach is potentially useful, it should be investigated before being used to assess the significance of resting state FC and it is likely that our simulation approach is more conservative. For studies investigating a *change* in connectivity between two or more conditions, computing the difference of connectivity values between conditions may negate the need for simulation. However, FC changes can also be affected by SNR (Schoffelen and Gross, 2009) and care must be taken in using such an approach.

### Investigating electrodynamic connectivity

The utility of MEG as a means to measure FC lies in its ability to assess the nature of electrodynamic connectivity. In this work we considered 4 different metrics for FC measurements, two based on envelope correlation, and two based on coherence. Our primary aim was to assess whether electrodynamic FC underlies fcMRI based metrics; envelope correlation is conceptually more similar to fcMRI than coherence based methodologies and results showed that correlation between left and right sensorimotor cortices peaks in the β frequency band (although significant correlation was also observed at higher frequencies (up to 40 Hz)). Interestingly, the time scale (Δ) on which connectivity is measured appears to be of some importance. Results in Fig. 3 show that *AEC* measured in 0.5 s time windows exhibits no significant correlation; significance increases with Δ and is maximum for a 10 s time window. Conversely, when computing *CAE*, segmenting envelopes into 10 s time windows resulted in significant inter-hemispheric correlation in the low β band only. Significance was increased as Δ was reduced and was maximal for Δ = 0.5 s. This provides information on the time scale of the correlation observed and these results are in agreement with those previously published by Liu et al. (2010) who, using channel space measurements, show that coherence between Hilbert envelope signals from opposite hemispheres peaks in the 0–0.1 Hz range. These analyses implicate a similar time scale to that of the fMRI BOLD response and this warrants further investigation. We should however state that as Δ is reduced, the reliability of correlation (and coherence) measurements is also reduced and care must be taken in interpretation of these results (see below). In general the CAE approach elicited higher correlation metrics than AEC; this is unsurprising since CAE effectively amounts to a low pass filter on the Hilbert envelope data, this elevates the signal to noise ratio and thus increase correlation coefficients measured.

Our inter-hemispheric measures of coherence and imaginary coherence between beamformer projected signals in the left and right sensorimotor areas yielded no values above our chosen 0.05 significance level. This could be to be due to the nature of coherence and the way in which it was measured. It is unlikely that electrical signals from two brain areas will remain coherent for long periods of time. Brain regions are more likely to exhibit transient coherence (Varela et al., 2001; Hadjipapas et al., 2004). In this work, we compute coherence averaged across a 300 s time window. It is therefore unsurprising that resulting values are small. Conversely our envelope correlation based approaches yielded much higher connectivity values (> 0.4 for *CAE*; > 0.08 for *AEC* compared to ~ 0.02 for *ICoh*). A likely reason is that envelope correlation approaches are less affected by noise, external interference and temporal jitter in MEG signals (this is particularly true of CAE), making them a more stable FC metric than coherence based approaches. Amplitude correlation metrics should not however be considered an improvement over coherence approaches since the latter are likely to be less sensitive to third party and common mode modulations. For example, given two systems with similar characteristic time scales, they are unlikely to appear as phase locked without being truly coupled. However, slower fluctuations in the amplitude may be caused by third party modulation or common mode effects. We therefore stress that envelope correlation and coherence based approaches are complementary and are likely to represent fundamentally different underlying physiological processes.

In Fig. 4 we show the time evolution of AEC and ICoh FC metrics and it is interesting to note a marked change in FC over 5 minute resting state recordings. Recently, interest has grown in dynamic FC measurements made using fcMRI (Chang and Glover, 2010) and interpreting large FC changes offers the potential for a better understanding of how these effects affect behavior. The current paper is limited since all FC measurements were made in the resting state, making FC change hard to interpret (see also below). However, Fig. 4 shows that dynamic FC measurements are possible using electromagnetic data; such measurements complement dynamic fcMRI metrics and this offers exciting opportunities to measure the time evolution of task induced change in FC.

Finally, there was some agreement between imaginary coherence and amplitude correlation metrics. Figs. 3 and 7 show that *ICoh* measurements peak in the β band, and at its maximum exceeds a threshold corresponding to p < 0.1. Fig. 4B shows correlation measured between dynamic *ICoh* and *AEC* metrics; with quantitative analysis showing that correlation is strongest in the β band. We might then speculate that amplitude correlation is driven by coherent bursts of synchronized activity in spatially separate cell assemblies. Since the imaginary part of coherence is implicated, this is known to exhibit a non-zero phase lag. In all *ICoh* measurements the absolute value of imaginary coherence was computed and no agreement was found without this step. The phase of coherent bursts may therefore change in different time windows leading to positive and negative imaginary coherence values. That said, correlation between ICoh and AEC timecourses was small (and did not reach statistical significance). It therefore remains likely that these measurements generate complementary information and that both should be considered in future studies.

### Cross-modal comparison

The cross hemisphere correlation results were supported by our cross modal comparison. The spatial agreement between FC measurements made using MEG and fMRI data is compelling. Results showed that the spatial signature of motor network connectivity can be measured independently using MEG and fMRI, and further that the location of peaks in correlation measured using fcMRI were similar to those measured using *CAE* applied to MEG data. (Note, *AEC* yielded similar peak positions, but due to the larger correlation coefficients observed, CAE results were presented.) Significant left hemisphere connectivity was observed in the θ, α, β and low γ frequency bands with the best spatial agreement in the β band. This is shown in Fig. 6 and supported by our quantitative analyses where spatial correlation between MEG and fcMRI derived FC maps is maximal in the β band. While some degree of overlap between FC measurements was observed for the α, β and low γ bands, the theta band showed FC that was spatially distinct from that in fcMRI. This could be a result of mislocalization (spatial filters are constructed independently for each frequency band and their accuracy depends on SNR. The θ band SNR is low potentially leading to mislocalization) or it could be indicative of a spatially distinct network mediated by θ oscillations. This warrants further investigation. It is unsurprising that β oscillations were most strongly implicated in motor cortex FC. Previous work (Salmelin et al., 1995; Stancak and Pfurtscheller, 1995; Pfurtscheller et al., 1996; Toma et al., 2000) has highlighted the role of β oscillations in the motor system and a close spatial relationship between β oscillations and the BOLD response has also been observed (Stevenson et al., 2011). Our results are in good agreement with other published work employing both concurrent EEG/fMRI and MEG (Mantini et al., 2007; Liu et al., 2010) and add further weight to a growing body of literature suggesting a close relationship between neural oscillations and BOLD.

The strong agreement between the spatial signature of FC measured using MEG and fcMRI acts to reduce confounds associated with either technique when used alone. The spatial accuracy of MEG is known to be limited by the ill posed inverse problem and the spatial similarity observed helps to validate the beamformer spatial filter approach. However, of more importance is validation of the observed BOLD correlations. Our data show that there is a sound electrophysiological basis for BOLD correlation between the left and right motor cortices. Such correlation could alternatively be due to one (or a combination) of the many possible sources of common mode influences on hemodynamics (for example, overlap of capillary beds, draining veins, pulsation and breathing). The fact that there is agreement in MEG suggests a neuronal and not a hemodynamic basis to fcMRI. It remains to be seen whether FC observed using fcMRI in other brain networks (e.g. the default mode, attention and salience networks) can also be substantiated using MEG.

### Limitations and future study

The measurement of functional connectivity using MEG is an interesting field that has potential to overcome many of the limitations of hemodynamic approaches. However, it remains a complex methodological problem and a complete solution is beyond the scope of a single paper. Here we present compelling evidence for the existence of electrophysiological FC, but these results must be taken at face value; they are valid within the limitations of the techniques used.

It is well known that the SNR of MEG changes with frequency and is low for frequencies in the high γ band. This means that the FC values computed could be affected since high correlation or coherence in the gamma band may be masked by poor SNR. Here signal to noise ratio peaked in the alpha and β range (e.g. in left motor cortex signal variance was 18 ± 4 nA m, 31 ± 9 nA m, 29 ± 6 nA m, 24 ± 5 nA m and 7 ± 1 nA m for the θ, α, low-β, high-β and the low-γ frequency ranges respectively). There was some demarcation between FC results and SNR since, while SNR was high in the α-band, there was little spatial agreement between fcMEG and fcMRI measurements (Fig. 7B). However, the fact that FC metrics will be more accurate in bands exhibiting high SNR remains a potential confound.

Despite the effectiveness of our simulations to test for spurious connectivity, some limitations remain. In our appendices we show that the beamformer effectively attenuates interference from a third brain source. However this was limited since only a single location for that source was chosen. It is conceivable that, had a different location been chosen, FC might have been affected. We also show effective attenuation of cardiac interference, however results presented do not prove categorically that the ECG or respiration has no effect on FC. In future studies, we advise measurement of physiological parameters (i.e. cardiac and respiratory cycles) alongside MEG. This would enable correlation of physiological parameters with MEG Hilbert envelope metrics in order to quantitatively assess the contribution of such interference. The simulation approach is flexible and it is possible to include interference sources in the simulation model; for example it may be possible to construct simulated data based on singular value decomposition of the real covariance matrix. However, this would rely on the explicit assumption that spatially separate sources exhibiting real FC would be represented by separate singular vectors in the SVD. An alternative approach would be to employ ICA; one might expect real functional networks and spurious networks elicited by interference to be separated by ICA, if they have an uncorrelated spatio-temporal signature. Judicious selection of independent components would thus eliminate spurious connectivity. These extensions should be the topic of future.

The way in which data were segmented for computation of FC should also be addressed. The reason for segmenting data was to investigate the time scale of connectivity and our results show similarity with previous work. The segmentation strategy also enabled us to examine the time evolution of FC and enabled comparison of envelope correlation and imaginary coherence FC metrics (Fig. 4). However there is bias in our segmentation strategy. For our AEC metric, the accuracy of the correlation coefficient is dependent on the time-frequency window in which the measurement is made, and the number of measurements averaged; error in the correlation coefficient is therefore dependent on the duration (Δ), the number of segments, *n*, and the bandwidth of the Hilbert envelope signal. For our Coh/ICoh metrics, coherence and imaginary coherence were computed within each segment (i.e. the cross-spectrum was measured for each segment and then averaged over segments). This technique is also known to exhibit bias for small data segments. Our real and simulated data are processed in the same way, and so differences between AEC, Coh and ICoh values for real and simulated data (Fig. 3) are not affected by bias due to data segmentation. However, Δ does change for separate rows in Fig. 3C and so, as stated above, the reader must exercise caution when interpreting the time scale of connectivity. For example, AEC for *Δ* = 0.5 s exhibits no significant results; this could mean that no correlation is observed on that time scale or it could result from poor estimation of correlation coefficients using data segmented within a 0.5 s window. Future work should aim to employ multi-taper spectral methods; these techniques do not rely on data segmentation and therefore do not exhibit the same bias as conventional the more spectral analysis used here.

Throughout this manuscript, we only measure resting state FC and this has proved interesting to compare MEG and fcMRI based metrics. However, future work should look to task driven changes in order to gain a more complete understanding of how FC changes with activity, and the relationship between metrics. For example, a direct comparison of the time evolution of imaginary coherence and envelope correlation between regions (as presented in Fig. 4) would be more meaningful if one knew that those brain areas were undergoing modulation. I.e. the peaks in the timecourses of FC (Fig. 4) could be better interpreted if they could be linked with a specific event (e.g. movement). However, care must be taken in using such measurements. Introducing a task will cause marked changes in cortical oscillatory power. Such changes, which occur in multiple brain areas, will illicit correlation between envelope signals from those areas. This would appear as an increase in FC, but could be due entirely to task driven change in separate unimodal brain regions. This is a limitation associated with all correlation approaches including those used in fcMRI. (Although such effects can be minimized by judicious selection of the time over which FC is measured (i.e. Δ).) In addition, changes in cortical oscillatory power cause changes in signal to noise ratio that can also result in spurious metrics of connectivity change (Schoffelen and Gross, 2009) (e.g. coherence measures become more reliable as SNR is increased and so an increase in SNR can appear as increased coherence, hence FC). Again such effects must be carefully considered in future studies examining task related FC changes.

Finally, the term ‘functional connectivity’ has been used to describe temporal correlation or coherence between signals from spatially separate brain areas. However, the fact that two signals are correlated does not necessarily mean that the brain areas from which they originate are functionally related. For example correlation could be driven by a third brain region and could be caused by changes in attention or arousal. In fact, work in monkey LFP (Leopold et al., 2003) concluded that envelope correlations most likely do not represent functional communication but common modulation due to spontaneous fluctuations in the arousal and attention systems. It is possible that the fluctuations observed in the present paper result from similar underlying mechanisms to those observed in monkey and this could be investigated further using task positive paradigms.

## Conclusion

In recent years, measurement of FC using fMRI has become a popular and important research area and to date has revealed the spatial signature of a number of hitherto unknown neural networks. However, the technique is fundamentally limited since fMRI is an indirect measure of brain activity. If the electrodynamic mechanisms underlying hemodynamic connectivity are to be elucidated, a multi-modal methodology will be key. In this paper, we have investigated *resting state FC* using fcMRI and MEG. We have shown that beamforming provides a suitable means to investigate FC in source space using MEG data. However, care must be taken when interpreting these measurements since cross talk between voxels in source space can potentially lead to spurious connectivity and this effect must be taken into account in all studies of this type. We have shown good spatial agreement between FC measured using MEG and fcMRI; FC between sensorimotor cortices was observed using both modalities, with the best spatial agreement when MEG data are filtered into the β band. This finding helps to reduce the potential confounds associated with each modality alone: while it helps reduce the uncertainties in spatial patterns generated by MEG (brought about by the ill posed inverse problem), addition of electrodynamic metric confirms the neural basis of fcMRI measurements. Finally, we have shown that multiple FC metrics can be applied to MEG data in order to investigate the nature of electrodynamic connectivity. Our results further those from previous studies and add weight to the argument that neural oscillatory processes are intimately related to both functional connectivity and the BOLD response. The clinical utility of MEG recordings has been highlighted in a number of recent papers (for example see Guggisberg et al., 2008; Stoffers et al., 2008) in which resting state measurements provide a protocol to differentiate patient groups. Here, we present a framework of doing source localization and FC measurement under resting state conditions. This could have great impact in future clinical work.

## Figures and Tables

**Fig. A1:**
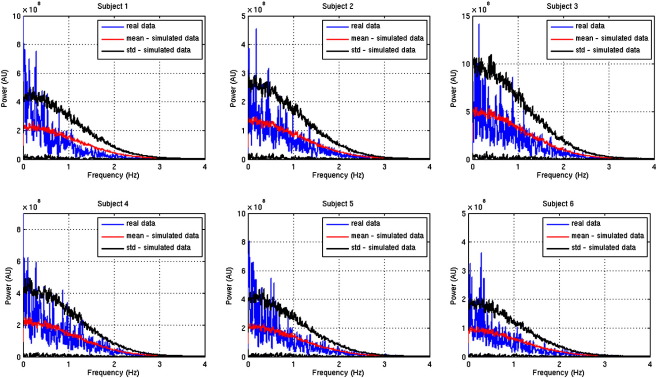
Hilbert envelope power spectra derived from real and simulated BFR MEG data for all six subjects. In all cases spectra are computed in the seed location in the left primary sensorimotor area.

**Fig. A2:**
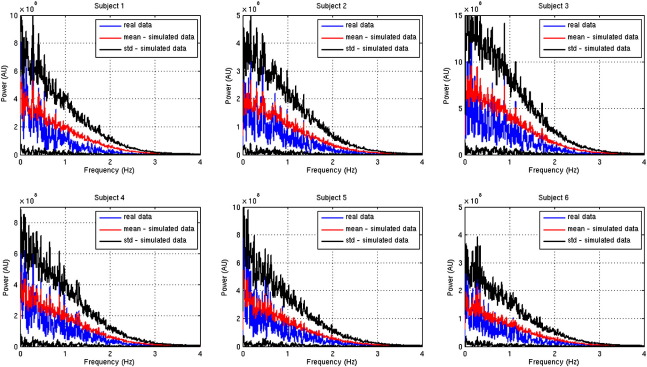
Hilbert envelope power spectra derived from real data, and simulated MEG data employing surrogate Hilbert envelopes. In all cases spectra are computed in the seed location in the left primary sensorimotor area.

**Fig. A3:**
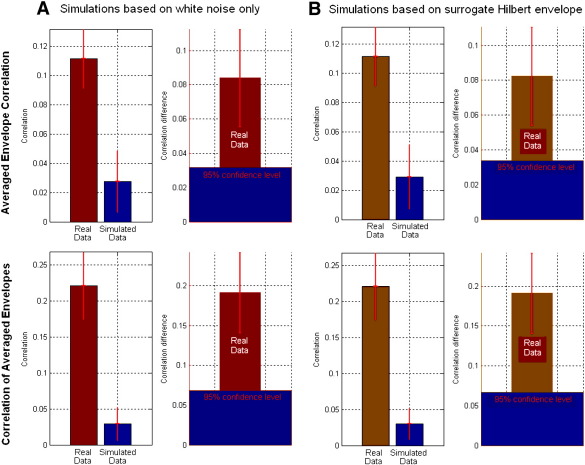
AEC (Δ = 10 s) (top panel) and CAE (Δ = 0.5 s) (bottom panel) computed in the β band. Within each of the four quadrants the left hand plot shows the raw FC metrics computed using real (red) and simulated (blue) data. In the right hand plot the red bar shows the corrected FC metric while the blue bar shows the correlation value required to reach statistical significance. A) Simulated MEG sources based on white noise (i.e. equivalent to the main manuscript). B) Simulated MEG sources based on a surrogate Hilbert envelope as explained above.

**Fig. A4:**
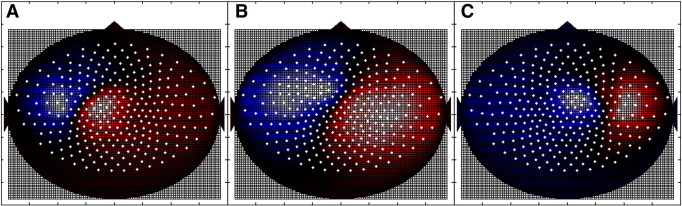
Fig. A4 Lead field patterns taken from a single representative source. A) Source in the left motor cortex. B) Interfering third source midway between the left and right motor cortices. C) Source in the right motor cortex.

**Fig. A5:**
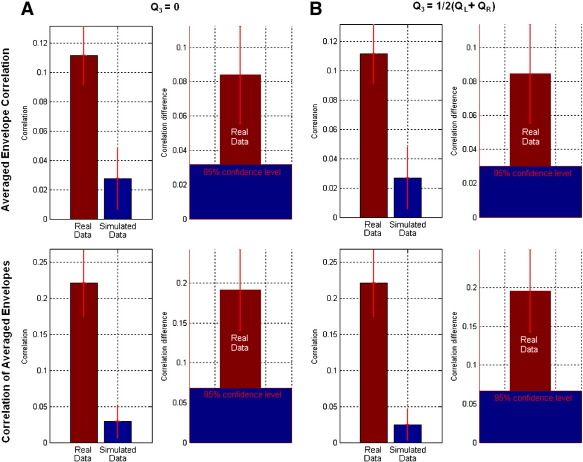
AEC (Δ = 10 s) (top panel) and CAE (Δ = 0.5 s) (bottom panel) computed in the β band. Within each of the four quadrants the left hand plot shows the raw FC metrics computed using real (red) and simulated (blue) data. In the right hand plot the red bar shows the corrected FC metric while the blue bar shows the correlation value required to reach statistical significance. A) The third source has zero amplitude (i.e. result equivalent to those in Fig. 3). B) Third source has amplitude equal to the mean of the amplitudes of the left and right motor cortex sources.

**Fig. A6:**
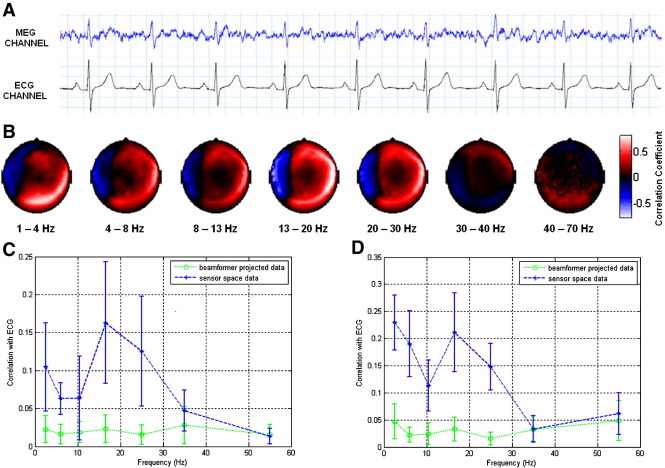
Rejection of cardiac interference with spatial filtering. A) The measured ECG and the magnetic field from a single MEG sensor. B) The channel space topography of cardiac interference plotted for each frequency band of interest. C) Correlation between MEG and ECG plotted as a function of frequency; the blue line shows correlation with sensors most affected by a source in left primary motor cortex; the green line shows correlation with the beamformer reconstructed timecourse for the same source in left motor cortex. D) Equivalent to (C) but shown for the right motor cortex. Notice the significant drop in ECG correlation with application of the spatial filter.

**Table 1:** Locations, in MNI coordinates, of the peaks in the left motor cortex identified using the fMRI localizer, the MEG localizer, the fcMRI analysis and the fcMEG analysis. Results for CAE in the β band are shown. All results are given as mean ± standard deviation across subjects.

Modality	x	y	z
fMRI-localizer	− 41 ± 4 mm	− 21 ± 8 mm	54 ± 6 mm
MEG-localizer	− 41 ± 3 mm	− 25 ± 6 mm	49 ± 9 mm
fcMRI	− 47 ± 7 mm	− 28 ± 7 mm	54 ± 5 mm
fcMEG—13–20 Hz	− 50 ± 9 mm	− 32 ± 5 mm	44 ± 9 mm
fcMEG—20–30 Hz	− 37 ± 6 mm	− 25 ± 5 mm	51 ± 6 mm

## Appendix 1 Frequency spectra of Hilbert envelope data

A possible limitation of our simulated data approach is that white noise is employed to generate the simulated seed and test timecourses. These white noise data are frequency filtered into bands of interest (α, β etc.) and the Hilbert envelope of the beamformer reconstructed source timecourses is used to test for spurious correlation. White noise by construction has no temporal structure whereas temporal structure may exist in Hilbert envelopes derived from real data. It is therefore possible that the differences in FC measured between the real and simulated data (Fig. 3) are not due to bivariate signal coupling between locations of interest, but are due to univariate temporal structure that exists in the genuine Hilbert envelope data that is not present in the simulated Hilbert envelope data.

To examine this in detail we first consider the frequency composition of the Hilbert envelopes, derived from real and simulated beamformer reconstructed (BFR) data. Fig. A1 shows BFR Hilbert envelope power spectra (HEPS) for all six subjects. The blue line shows the HEPS derived from real data; the red line shows HEPS for simulated data (averaged over all iterations of the simulations); the black lines show the averaged simulated HEPS plus/minus the standard deviation computed across iterations. Data show HEPS derived in the low-β band only. While the simulated spectra are a reasonable match for spectra derived from real data, it is interesting to note that the two measurements diverge in the 0 Hz–0.5 Hz range; this is particularly apparent for subjects 1, 5 and 6. This divergence potentially indicates temporal structure in the real envelope that is not present in the simulated envelope data and therefore warrants further investigation.

In order to test whether the differences in FC measurement between real and simulated data, observed in Fig. 3, are due to bivariate coupling, or are a result of the low frequency differences between the real and simulated spectra shown in Fig. A1, we recomputed a new set of simulated MEG data. These new simulations were undertaken for the β band only and were based on a two step process:

1) *Construction of a surrogate envelope:* The Hilbert envelope from real data was computed and Fourier transformed to give.
  (A1) $$H_{F}\left(f\right)=A\left(f\right)\text{exp}(i\phi \left(f\right))$$
  Where *H*_*F*_(*f*) represents the Fourier transformed Hilbert envelope; *A*(*f*) represents the amplitude of each frequency component and *ϕ*(*f*) represents the phase of each frequency component. Surrogate envelope data were then computed in frequency space as:
  (A2) $$H_{\mathrm{FSurrogate}}\left(f\right)=A\left(f\right)\text{exp}\left(i\phi_{\mathrm{new}}\left(f\right)\right)$$
  Where *ϕ*_*new*_ is a random number between − π and π. Maintaining the amplitude of frequency components ensures that the surrogate Hilbert envelope has the same power spectrum as that derived from real data. However, phase randomization ensures that the temporal profile of the surrogate Hilbert envelope is not correlated with that of the real Hilbert envelope.
2) *Construction of the simulated dataset:* White noise data were generated and frequency filtered into the β band (as previously) to yield a simulated source timecourse. This is then multiplied (element by element) with the surrogate Hilbert envelope. Multiplication in this way ensures that the temporal structure of the simulated source envelope is similar to that of a real source envelope. Simulated source timecourses were multiplied by the lead fields and real MEG noise added in order to construct simulated datasets. Both the white noise and the phase randomization were different for each iteration of the simulation.

Simulated data were projected through the same spatial filters as those used to project the real data, resulting in beamformer reconstructions of the simulated left and right motor cortex sources. Simulated reconstructed sources were then Hilbert transformed to derive the BFR Hilbert envelopes. Fig. A2 shows the BFR HEPS derived from real data (blue) and our newly simulated data (red line). The black lines show plus/minus standard deviation for the simulated data. Notice now that the HEPS derived from real data are better encompassed by the new simulated spectra than in the original case (Fig. A1) where simulated sources were based on white noise only.

Connectivity between the seed and test locations was measured using both the averaged envelope correlation (AEC) (Δ = 10 s) and the correlation of averaged envelope (CAE) (Δ = 0.5 s) techniques. Fig. A3 shows results of both metrics in the β band; original FC values (computed with white noise simulated data) are compared to the modified FC values (computed using the surrogate Hilbert envelope simulations).

As shown in Fig. A3, simulating MEG sources based on surrogate Hilbert envelopes had little effect on the overall FC result. AEC and CAE values computed in simulation showed a small increase using a surrogate envelope approach, however this effect was small and correlation between signals in the left and right motor cortices remains significant. The outcome of these simulations implies that the β band effects observed in the manuscript are due to bivariate coupling between locations of interest, and are not a result of differences in the temporal structure of real and simulated timecourse data.

## Appendix 2 The existence of an interfering brain source

The simulation approach that we employ to test for spurious connectivity does not represent a perfect solution to eliminate all spurious connectivities. It is possible that a third brain source (not accounted for by our simulations) may interfere with both the seed and test sources and thus cause spurious connectivity between them. In order to examine the effects of such interference, here we undertake an extension to the simulations described above.

As previously, dipolar sources were simulated at the seed and test locations in the right and left motor cortices respectively. These sources had equivalent orientation and amplitude to those measured using real data; however, their timecourses were random and uncorrelated. In addition, a third source was added: The location of the third source was the mid-point of a vector joining the left and right motor cortices. This was computed on a subject-by-subject basis and chosen since it was the most likely location for a source that would interfere with both locations of interest. The orientation of the third source was set according to that measured using real data (computed on a subject-by-subject basis). The lead fields for all three sources are shown in Fig. A4. The third source timecourse was not correlated with either of the other simulated sources. The amplitude of the third source was varied: in the first case it was set to zero (to give a result equivalent to that in Figs. 3 and A7); in the second case it was set as the mean measured amplitude of the left and right motor cortex sources. Simulated MEG data were added to genuine noise data in order to construct the simulated datasets. Note that all measurements were limited to the low β frequency band.

Simulated data were projected through the same spatial filters as those used to project the real data resulting in beamformer reconstructions of the simulated left and right motor cortex sources. Connectivity between the seed and test locations was measured using both AEC (Δ = 10 s) and CAE (Δ = 0.5 s). Results are shown in Fig. A5.

Results in Fig. A5 show that a source placed midway between the left and right motor cortices has little effect on the FC values measured in simulation. The implication is that the beamformer spatial filters (derived from the real data) act as an effective means to suppress this third source.

## Appendix 3 The existence of an interfering non-brain source

In a single subject, the electrocardiogram (ECG) was acquired concurrently with MEG data by placing three electrodes on the subject's chest. ECG data were acquired specifically to assess the contribution of electrical interference from the heart to the MEG. In order to assess the effect of non-neuronal physiology on raw and beamformer projected MEG data, the level of cardiac interference in a single subject was measured. The ECG was filtered into the same frequency bands as those used for MEG analysis. The Pearson correlation coefficient between the filtered ECG and the filtered MEG was assessed at each MEG sensor, resulting in a map showing the topographical distribution, in sensor space, of cardiac interference for each of the seven frequency bands. Note that Pearson correlation values were computed for the data acquired during the resting state phase of the experiment only. MEG data were then projected into the brain. (To ensure optimized spatial resolution, the beamformer weights were based on covariance computed using the entire dataset.) Beamformer projected timecourses were extracted from two locations of interest in the left and right sensorimotor areas (as defined by the MEG localizer experiment) and the Pearson correlation coefficients between the ECG and the two projected timecourses (again for the resting phase of the experiment) were computed. These correlation values were compared to equivalent values computed at the MEG sensors most affected by the motor cortex sources (i.e. those MEG sensors with a lead field greater than 80% of the maximum absolute lead field at any sensor).

Fig. A6 shows an example of the interference rejection properties of the beamformer. As alluded to in the introduction, a confound of fcMRI is that results can be affected by non-neuronal physiological interference caused by, for example, the cardiac cycle, changes in the cardiac cycle, respiration or changes in respiration rate. Here we show that MEG can also be affected by similar confounds since it is susceptible to interference from non-neuronal sources, in this case the electrical signal from the heart. Fig. A6A shows the ECG plotted alongside the MEG signal from a single sensor. Fig. A6B shows the Pearson correlation between the frequency filtered ECG and the frequency filtered channel space MEG signals. Note that cardiac interference affects a large number of MEG sensors and, unless adequately dealt with, could lead to spurious connectivity measurement, particularly when using channel space metrics. Figs. A6C and D highlight the artifact rejection properties of the beamformer. In both cases the blue line shows Pearson correlation between the ECG and the MEG sensors most affected by sources in the left (C) and right (D) sensorimotor cortices. The green line shows correlation between the ECG and the beamformer reconstructed timecourses from the peak voxel of interest in the left (C) and right (D) sensorimotor cortices. Notice that for sensor space data, high correlation with the ECG is observed, and further that correlation is inhomogeneous with respect to frequency. However, following application of the spatial filter, correlation is significantly reduced and is less than 0.05 in all frequency bands.

## Appendix 4 The existence of an interfering brain source

In Fig. 3C, AEC, CAE, Coh and ICoh measurements extracted from real data have been corrected by subtraction of the equivalent metrics applied to simulated data. In Fig. A7, for completeness, we show the AEC, CAE, Coh and ICoh metrics applied to real and simulated data separately. In all cases the black line shows the metrics applied to simulated data while the blue curve shows the metrics applied to real data. The four columns show the four separate FC metrics and the 5 rows show 5 different values of delta.
